# TRIM56 enhances adenoviral E1A steady state to improve oncolytic adenovirus therapy efficacy

**DOI:** 10.1128/jvi.00041-25

**Published:** 2025-06-03

**Authors:** Nan Sun, Jikai Zhang, Chen Zhang, Hang Yin, Botao Zou, Yunfeng Geng, Wanjun Gao, Yuxin Zhang, Gang Wang, Junnian Zheng, Lin Fang

**Affiliations:** 1 Cancer Institute, Xuzhou Medical University 38044 , Xuzhou, Jiangsu, China; 2 Center of Clinical Oncology, Affiliated Hospital of Xuzhou Medical University 529858 , Xuzhou, Jiangsu, China; 3 Jiangsu Center for the Collaboration and Innovation of Cancer Biotherapy, Cancer Institute, Xuzhou Medical University 38044 , Xuzhou, Jiangsu, China; 4 Department of Pathogen Biology and Immunology, Biology and Immunology, Jiangsu Key Laboratory of Immunity and Metabolism, School of Basic Medical Sciences, Xuzhou Medical University 666893 , Xuzhou, Jiangsu, China; 5 Department of Oncology, The First People’s Hospital of Yancheng, Yancheng, Jiangsu, China; International Centre for Genetic Engineering and Biotechnology, Trieste, Italy

**Keywords:** TRIM56, E1A, STING, adenoviruses, oncolytic viruses

## Abstract

Oncolytic adenoviruses (OAVs) engineered to carry antitumor or immune-activating transgenes are being actively explored for cancer immunotherapy. While many efforts have focused on modifying OAVs to counteract the immunosuppressive tumor microenvironment, comparatively less attention has been given to enhancing OAV replication by targeting host factors to increase oncolytic activity. Here, we report that TRIM56 is strongly upregulated during HAdV-C5 infection, with its expression correlating with increasing levels of the viral E1A protein, ultimately promoting HAdV-C5 replication. Mechanistically, TRIM56 stabilizes the viral E1A protein and enhances viral genome transcription. To leverage this effect, we engineered a recombinant oncolytic adenovirus expressing TRIM56 (OAV-TRIM56) and found that it achieved significantly higher replication titers *in vitro* compared to conventional OAVs, leading to superior antitumor efficacy *in vivo*. Our study presents a novel strategy to enhance OAV replication by targeting host factors, offering a promising approach for improving oncolytic virotherapy.

**IMPORTANCE:**

Adenoviruses (Ads) can be engineered into replication-defective adenoviral (Adv) vectors and replication-competent oncolytic adenovirus (OAd), both of which are widely used in gene therapy and virotherapy. Understanding the mechanisms regulating adenoviral infection is crucial for optimizing the therapeutic potential of Adv and OAd. In this study, we demonstrate for the first time that TRIM56, a host protein broadly expressed in various cell types, stabilizes the adenoviral E1A protein and assists E1A in antagonizing STING, thereby significantly enhancing adenoviral replication. Our findings provide new insights into strategies for improving the efficacy of Adv and OAd in gene therapy.

## INTRODUCTION

Human adenoviruses (HAdVs) can cause gastroenteritis, conjunctivitis, and respiratory and urinary tract infections, but generally do not cause severe clinical symptoms (1, 2). However, in immunocompromised individuals, HAdV infections can result in severe morbidity (3). To date, no effective treatment exists for HAdV infections (4). HAdVs have been investigated for 70 years, and many of their characteristics have been well identified. Some studies have explored the interactions between HAdV and host factors, as well as antiviral immunity *in vivo*. The PDZ2 domain of MAGI-1 inhibits adenovirus infection by facilitating proteolysis of the adenovirus receptor (5); Mind Bomb 1 regulates adenoviral genome release at the nuclear pore complex (6). Nevertheless, more important molecular mechanisms likely remain to be explored.

In recent years, adenoviruses have emerged as one of the most widely used viral vectors in clinical gene therapy, including applications in oncolytic therapy and vaccination. The development and clinical application of oncolytic viruses have gained significant attention, with adenoviruses being among the most extensively studied viral platforms for oncolytic virotherapy (7). Adenoviruses function as oncolytic agents by inducing localized inflammation, viral replication, disruption of the tumor microenvironment, and the release of tumor antigens, ultimately triggering a systemic antitumor immune response that may lead to complete tumor eradication (8). Therefore, understanding host immune responses to viral infections and optimizing adenoviral vectors for therapeutic applications, particularly in cancer treatment, are critical for advancing both fundamental research and clinical translation.

Gene therapy based on the HAdV vector has broad application prospects (9, 10). Considerable efforts have been dedicated to modifying the natural properties of HAdV to enhance its utility in gene transfer, oncolytic virotherapy, and vaccine development. ONYX-015 (dl1520) was the first oncolytic adenovirus to enter clinical trials for head and neck cancer treatment. Building on this, the Oncorine variant (H101), developed through partial deletion of the adenovirus type 5 genome and the E3 gene, has been approved for clinical application in China (11, 12). In addition, DNX-2401 (Delta-24-RGD), the first Ad Delta-24 derivative to enter clinical trials, is primarily used for glioma treatment and has completed four Phase I trials (NCT01582516, NCT01956734, NCT02798406, NCT02197169) (12).

Despite promising preclinical results, several key challenges remain in the clinical application of oncolytic adenoviruses, including limited oncolytic efficacy, premature viral clearance, and insufficient activation of tumor-specific immune responses (8). Among these challenges, tumor-selective viral replication is essential for both direct oncolysis and the subsequent induction of antitumor immunity. Enhancing the replication capacity of oncolytic adenoviruses is therefore critical for improving their therapeutic efficacy. A deeper understanding of HAdV biology, particularly its infection dynamics and immune interactions, will be instrumental in advancing HAdV-based gene therapy and virotherapy.

Numerous studies have shown that the TRIM protein family plays a crucial role in regulating viral replication, often by inhibiting the replication of viruses such as yellow fever virus, dengue virus, Zika virus, and influenza A virus (13–16). In addition, TRIM proteins modulate innate immune signaling pathways to enhance host antiviral responses (17). The TRIM family consists of approximately 80 distinct members in humans (18) and is characterized by a conserved RBCC (RING-B-box-coiled-coil) domain structure. These proteins are categorized into subfamilies (C-I to C-XI) based on variations in their C-terminal domains (19, 20).

TRIM56, also known as ring finger protein 109 (RNF109), is an 81 kDa protein composed of 755 amino acids, encoded by a gene located on human chromosome 7(18). As a member of the C-V TRIM subfamily, TRIM56 lacks a C-terminal and contains three core domains: a RING domain, a B-box domain, and a coiled-coil domain (21). TRIM56 has E3 ubiquitin ligase activity and has been shown to inhibit several positive-sense RNA viruses, including members of the Flavivirus family (yellow fever virus, dengue virus, and bovine diarrhea virus) and human coronavirus (OC43) (22). As an E3 ligase, TRIM56 relies on its RNA-binding activity to act as a host restriction factor against Zika virus (15). In addition, TRIM56 suppresses hepatitis B virus (HBV) infection and replication by inhibiting the HBV core promoter (23). Its antiviral effects against influenza A and B viruses are mediated through its C-terminal tail, which blocks viral RNA synthesis (14). However, our study suggests that TRIM56 plays a distinct and previously uncharacterized role during HAdV-C5 infection.

Here, we report that TRIM56 is significantly upregulated during HAdV-C5 infection and plays a crucial role in promoting viral replication. Contrary to its well-established antiviral functions, our findings reveal a unique and unexpected role for TRIM56 in facilitating adenoviral replication. Mechanistically, TRIM56 interacts with the adenoviral E1A protein, stabilizing it and working synergistically to enhance viral replication. To leverage this function, we engineered a recombinant oncolytic adenovirus OAV expressing TRIM56 (OAV-TRIM56) to enhance oncolytic virus replication. Compared to conventional OAVs, the TRIM56-expressing OAV achieves significantly higher replication titer *in vitro* and exhibits stronger antitumor activity *in vivo*. Our findings provide novel insights into the role of host factors in OAV replication and offer a promising strategy for improving the therapeutic efficacy of oncolytic virotherapy.

## RESULTS

### TRIM56 promotes HAdV replication

**Fig 1:**
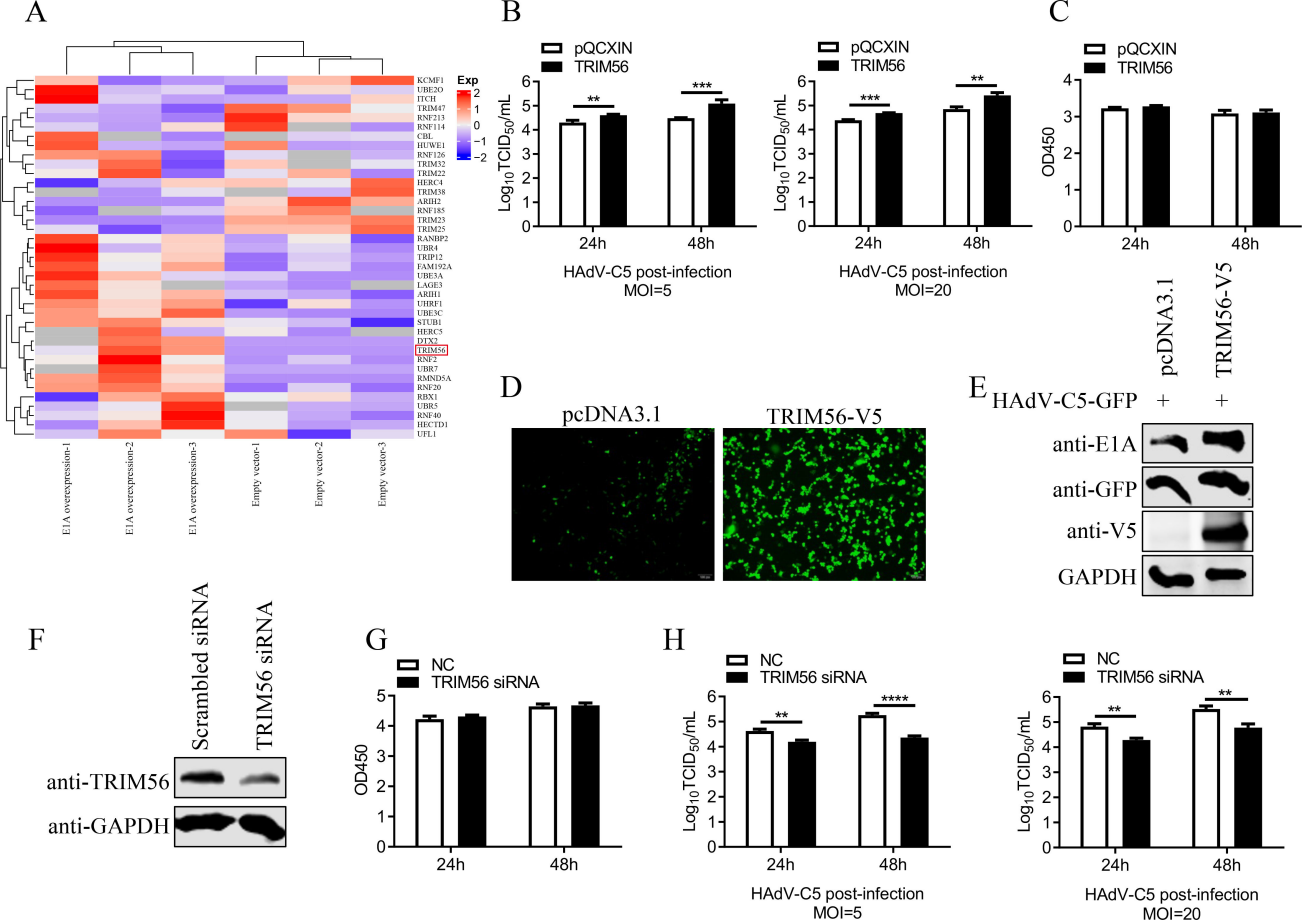
TRIM56 promotes HAdV-C5 replication. (**A**) Results of mass spectrometry analysis in E1A overexpression cells and the control cells. The mass spectrometry results were clustered according to E3 ubiquitin ligase. (**B**) An A549 cell line stably overexpressing TRIM56 was established. HAdV-C5 was used to infect TRIM56 stably overexpressing and control A549 cells at MOIs of 5 and 20, respectively. The supernatant was collected at the indicated times, and TCID50 assays were used to detect the virus titer in HEK293 cells. **P < 0.01; ***P < 0.005. (**C**) A CCK-8 assay was used to detect the viability of A549 cells overexpressing TRIM56. (**D and E**) A549 cells infected with the HAdV-C5-GFP virus for 24 h at an MOI of 10 were observed via fluorescence microscopy. Scale bar, 200 µm. Then, western blot analysis of virus-infected cells was performed using anti-GFP and anti-E1A antibodies. (**F**) TRIM56 expression in A549 cells was knocked down by siRNA. After A549 cells were transfected with siRNAs targeting TRIM56 or control siRNA for 48 h, whole-cell lysates were collected and analyzed via western blotting with a rabbit anti-TRIM56 antibody. (**G**) A CCK-8 assay was used to detect the effect of siRNA targeting TRIM56 or control siRNA on the viability of A549 cells. (**H**) siRNAs targeting TRIM56 and the control siRNA were transfected into A549 cells for 36 h, after which HAdV-C5 virus at an MOI of 5 or 20 was used to infect the cells. Then, the virus supernatant was collected after infection at 24 or 48 h, and the virus titer in HEK293 cells was determined via TCID50 assays. **P < 0.01; ****P < 0.0001. At least three independent experiments were conducted.

Mass spectrometry analysis revealed a significant increase in TRIM56 expression in E1A-overexpressing cells compared to control cells (Fig. 1A). Based on this, we hypothesized that TRIM56 may regulate HAdV infection. To investigate its role in the viral life cycle, A549 cells overexpressing TRIM56 were infected with HAdV-C5 at a multiplicity of infection (MOI) of 5 or 20. TRIM56 overexpression significantly promoted viral replication, with a 10-fold increase in virus titers at 48 hours post-infection (hpi) compared to control cells at an MOI of 5 (Fig. 1B). CCK-8 assays confirmed that TRIM56 overexpression did not impact cell viability (Fig. 1C). Infection with HAdV-C5-GFP virus (HAdV-C5-GFP) further showed that TRIM56 overexpression increased both the number of infected cells and fluorescence intensity (Fig. 1D). Western blot analysis demonstrated a significant increase in E1A viral protein and GFP expression in TRIM56-overexpressing cells (Fig. 1E).

To assess the effect of TRIM56 depletion, A549 cells were treated with TRIM56-specific siRNA (Fig. 1F). CCK-8 assays indicated that TRIM56 knockdown did not affect cell viability (Fig. 1G). When infected with HAdV-C5, TRIM56-knockdown cells exhibited a 10-fold reduction in virus titers at 48 hpi with an MOI of 5 (Fig. 1H). These results collectively indicate that TRIM56 positively regulates HAdV-C5 replication and enhances progeny virus production.

### TRIM56 promotes transcription of HAdV-C5 genes

**Fig 2:**
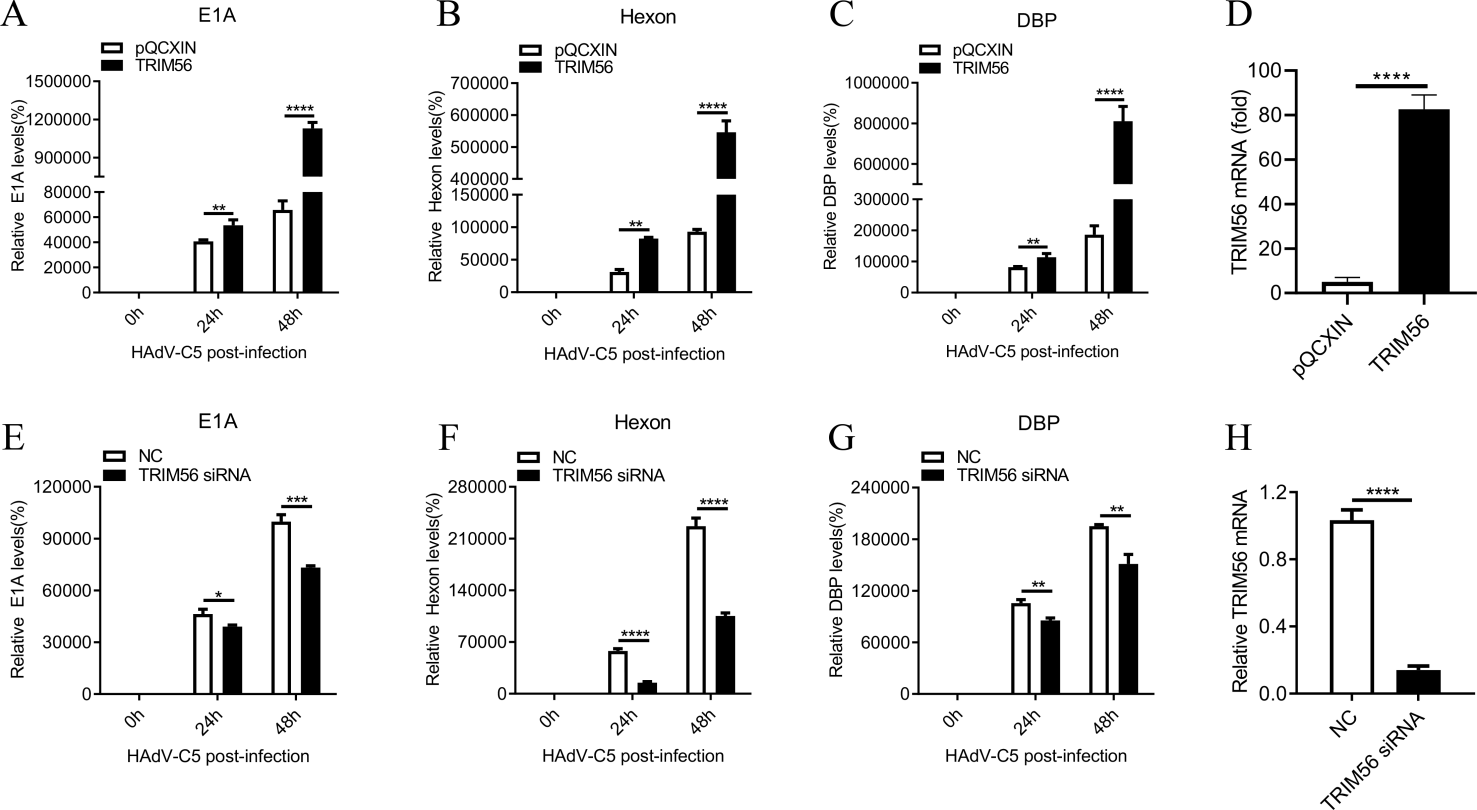
TRIM56 promotes the transcription of HAdV-C5 genes. (A, B, and C) HAdV-C5 with an MOI of 20 infected TRIM56 stably overexpressing or control A549 cells. Whole cells were collected after infection at 24 h or 48 h, after which the RNA levels of the E1A, DBP, and Hexon transcripts were analyzed via RT-qPCR. The means and standard deviations (SDs) of three independent experiments were normalized. **P < 0.01; ****P < 0.0001. (**D**) RT-qPCR confirmed that TRIM56 was stably overexpressed. ****P < 0.0001. (E, F, and G) The HAdV-C5 virus was used to infect A549 cells treated with siRNA targeting TRIM56 or control siRNA for 36 h at an MOI of 20. Then, whole cells were collected after infection at 24 h or 48 h, and the RNA levels of the E1A, DBP, and Hexon transcripts were analyzed via RT-qPCR. The results are presented as the means and SDs of three experiments, which were normalized to the level of GAPDH. *P < 0.05; **P < 0.01; ***P < 0.001; ****P < 0.0001. (**H**) RT-qPCR confirmed that TRIM56 was significantly knocked down in A549 cells via siRNA targeting TRIM56. ****P < 0.0001. At least three independent experiments were conducted.

Since TRIM56 enhances viral replication, we examined whether it also influences viral gene transcription and genome replication. A549 cells overexpressing TRIM56 were infected with HAdV-C5, and qRT-PCR was used to assess the transcript levels of E1A, Hexon, and DBP. Compared to control cells, TRIM56-overexpressing cells exhibited significantly higher transcript levels of these viral genes at both 24 and 48 hpi (Fig. 2A through C). Real-time PCR confirmed the elevated expression of TRIM56 in TRIM56-overexpressing cells (Fig. 2D).

Conversely, TRIM56 knockdown significantly reduced E1A, Hexon, and DBP transcript levels in HAdV-C5-infected cells (Fig. 2E, F, and G). Real-time PCR confirmed reduced TRIM56 expression in siRNA-treated cells (Fig. 2H). Collectively, these results indicate that TRIM56 overexpression enhances viral gene transcription, whereas its knockdown suppresses it.

### TRIM56 interacts with E1A

**Fig 3:**
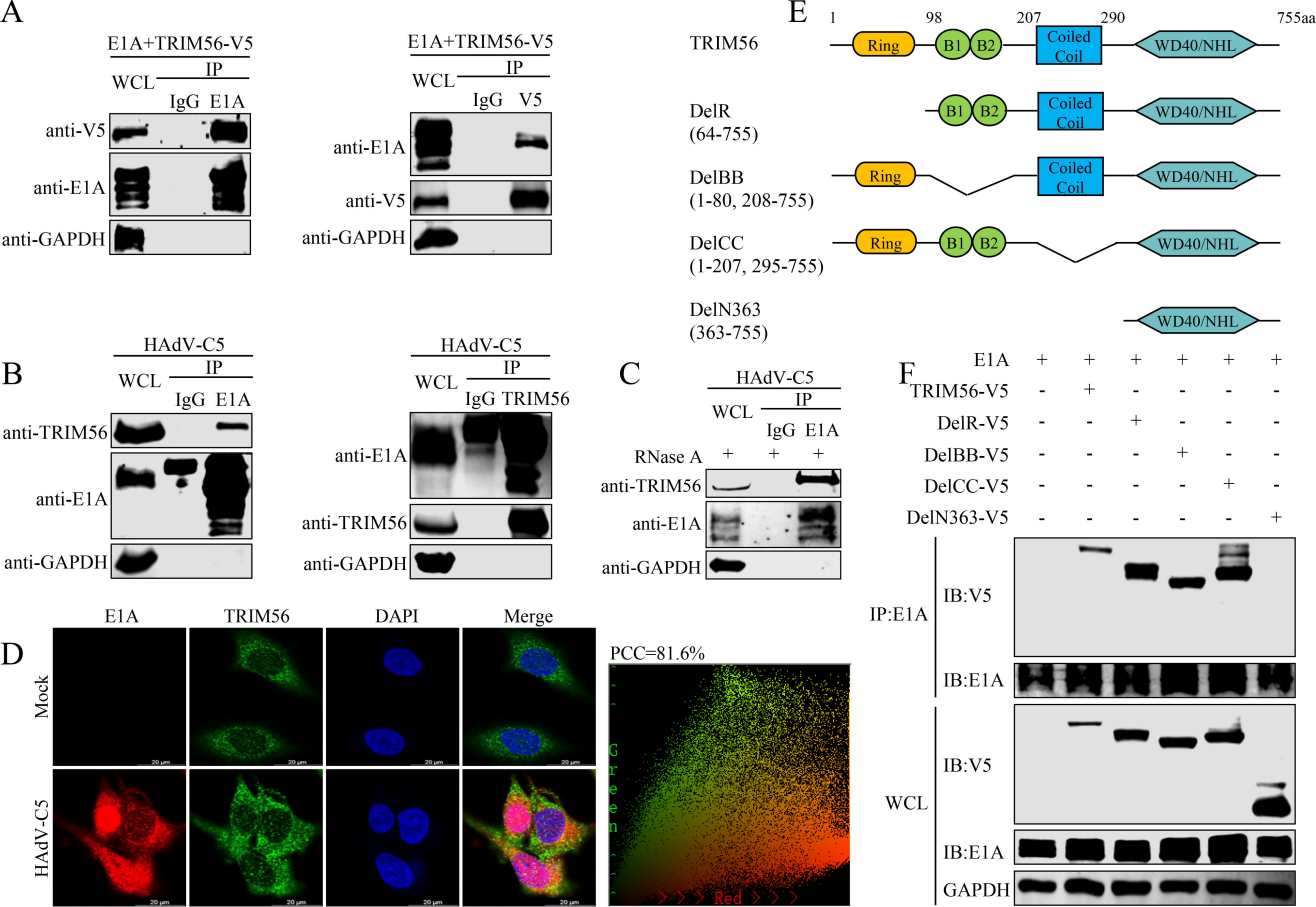
TRIM56 interacts with E1A. (**A**) V5-tagged TRIM56 and E1A from HAdV-C5 were cotransfected into HEK293T cells for 36 h. The whole-cell lysates were immunoprecipitated (IP) via an anti-E1A antibody (left panel), an anti-V5 antibody (right panel), or control IgG, and western blotting was performed as indicated. (**B**) HAdV-C5 was used to infect A549 cells at an MOI of 20 for 30 h. Then, the cells were collected, and whole-cell lysates were immunoprecipitated with anti-E1A antibody (left panel), anti-TRIM56 antibody (right panel), or control IgG, and western blotting was performed. (**C**) A549 cells were infected at an MOI of 10 with HAdV-C5 for 30 h. Cell lysates were treated with RNase A and then immunoprecipitated with anti-E1A antibody or control IgG. (**D**) HAdV-C5-infected A549 cells with an MOI of 20 for 14 h to observe the co-localization of viral E1A and TRIM56. The cells were fixed using 4% paraformaldehyde, incubated with anti-E1A and anti-TRIM56 antibodies, then analyzed by confocal microscopy. The Pearson correlation coefficient of E1A (red) and TRIM56 (green) was shown on the right panel. PCC: Pearson correlation coefficient. Scale bar, 20 µm. (**E**) Map of TRIM56 truncation mutants. (**F**) HEK293T cells were cotransfected with E1A and various V5-tagged TRIM56 mutants for 36 h. The cells were collected, and the whole-cell lysate was immunoprecipitated with an anti-E1A antibody. At least three independent experiments were conducted.

To determine whether TRIM56’s role in HAdV-C5 replication is linked to its interaction with E1A, co-immunoprecipitation (co-IP) assays were performed. HEK293T cells cotransfected with V5-tagged TRIM56 and E1A from HAdV-C5 demonstrated a specific interaction between TRIM56 and full-length E1A (Fig. 3A; Fig. S1). In addition, endogenous TRIM56 interacted with viral E1A during HAdV-C5 infection (Fig. 3B). RNase A treatment did not affect this interaction (Fig. 3C), suggesting that the TRIM56-E1A interaction is independent of RNA. Immunofluorescence assays revealed that E1A colocalized with TRIM56 in the cytoplasm during HAdV-C5 infection (Fig. 3D). V5-tagged TRIM56 truncation mutants (DelR, DelBB, DelCC, and DelN363) were generated to map the interaction region with viral E1A protein (Fig. 3E). Coimmunoprecipitation experiments revealed that DelN363 was unable to interact with E1A, indicating that the N-terminal region of TRIM56 is required for E1A binding (Fig. 3F). These results together indicate that there is a specific interaction between TRIM56 and E1A *in vivo* and *in vitro*, which, in turn affects adenovirus replication.

### TRIM56 induces higher expression of the viral E1A of HAdV-C5

**Fig 4:**
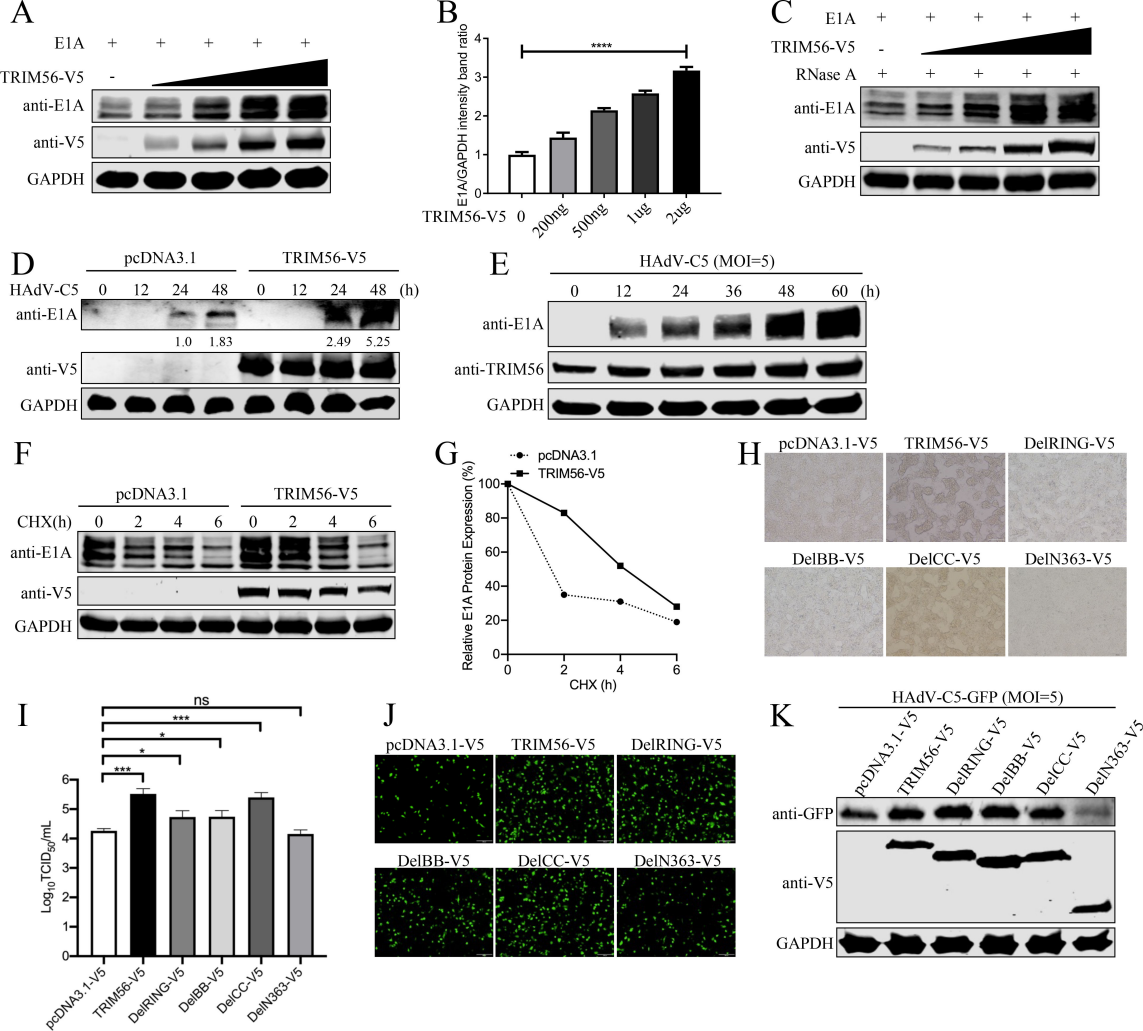
TRIM56 stabilizes the E1A protein. (**A**) Increased levels of both TRIM56-V5 and E1A were cotransfected into HEK293T cells for 36 h. Cell lysates were immunoblotted with anti-E1A and anti-V5 antibodies. (**B**) Quantification of relative E1A levels of three independent experiments in (**A**). ****P < 0.0001. (**C**) HEK293T cells were cotransfected with increased TRIM56-V5 and E1A. Cell lysates were treated with RNase A and then subjected to western blot analysis with anti-E1A antibody. (**D**) A549 cells transfected with TRIM56-V5 or empty vector plasmid were infected with HAdV-C5 at an MOI of 5. The cells were collected at 0, 12, 24, and 48 h post-infection and subjected to immunoblotting with anti-E1A and anti-V5 antibodies. (**E**) HAdV-C5 was used to infect A549 cells at an MOI of 5. The whole-cell lysate was collected at 0, 12, 24, 36, 48, and 60 h post-infection, and western blotting was conducted using anti-E1A and anti-TRIM56 antibodies. (**F and G**) HEK293T cells were cotransfected with TRIM56-V5 or pcDNA3.1, together with E1A, respectively. The cells were treated with 20 µg/mL cycloheximide for 0, 2, 4, or 6 h, and western blot analysis was performed with the indicated antibodies. Quantification of relative E1A levels is shown in (**G**). (**H and I**) V5-tagged TRIM56 and its various mutants were infected with HAdV-C5 at an MOI of 5. The cytopathic effect after 30 h post-infection was observed by microscope (**H**). Then the supernatant was collected, and the virus titer was determined using the TCID50 assay on HEK293 cells (**I**). Scale bar, 500 µm. All experiments were repeated at least three times. (**J and K**) V5-tagged TRIM56 and its various mutants transfected cells were infected with HAdV-C5-GFP virus at an MOI of 5. The virus-infected cells after 30 h post-infection were observed using a fluorescence microscope (**J**). Western blot was performed to detect the GFP protein using an anti-GFP antibody (**K**). Scale bar, 200 µm. At least three independent experiments were conducted.

Next, we investigated whether TRIM56 affects the expression of E1A from HAdV-C5 through dose-dependent experiments. The results showed that the expression of E1A increased with increasing TRIM56 expression (Fig. 4A; Fig. S2). Furthermore, quantification of the western blots revealed that the expression of E1A increased to the maximum value, while the expression of TRIM56 was the highest (Fig. 4B). This effect was RNA-independent (Fig. 4C). Time-course experiments showed that TRIM56-overexpressing cells exhibited sustained E1A expression, particularly at 48 hpi (Fig. 4D). Western blot analysis of A549 cells infected with HAdV-C5 at different time points (0, 12, 24, 36, 48, and 60 h) demonstrated a consistent increase in endogenous TRIM56 and E1A protein levels over time (Fig. 4E). Cycloheximide chase assays further confirmed that TRIM56 stabilizes E1A protein (Fig. 4F and G). Collectively, these results suggest that TRIM56 promotes the stability of E1A independent of RNA.

Subsequently, we explored the specific functional domains of TRIM56 that promote HAdV-C5 replication. HEK293T cells overexpressing V5-tagged TRIM56 and its truncation mutants (DelR, DelBB, DelCC, and DelN363) were infected with HAdV-C5. The results revealed that the DelN363 mutant lost the ability to promote HAdV-C5 replication, while the other mutants could significantly promote HAdV-C5 replication (Fig. 4H and I). In addition, HAdV-C5-GFP virus (HAdV-C5-GFP) infected A549 cells overexpressing V5-tagged TRIM56 and its truncation mutants (DelR, DelBB, DelCC, and DelN363). The results showed that there was no difference in the fluorescence intensity of virus-infected cells when the DelN363 mutant was overexpressed compared to the control cells (Fig. 4J). Western blot suggested that the GFP protein in DelN363 mutant overexpressing cells was less than other mutants but similar to the control cells (Fig. 4K). These findings indicate that TRIM56 stabilizes E1A and enhances HAdV-C5 replication through its N-terminal region.

### TRIM56 facilitates STING degradation by E1A

**Fig 5:**
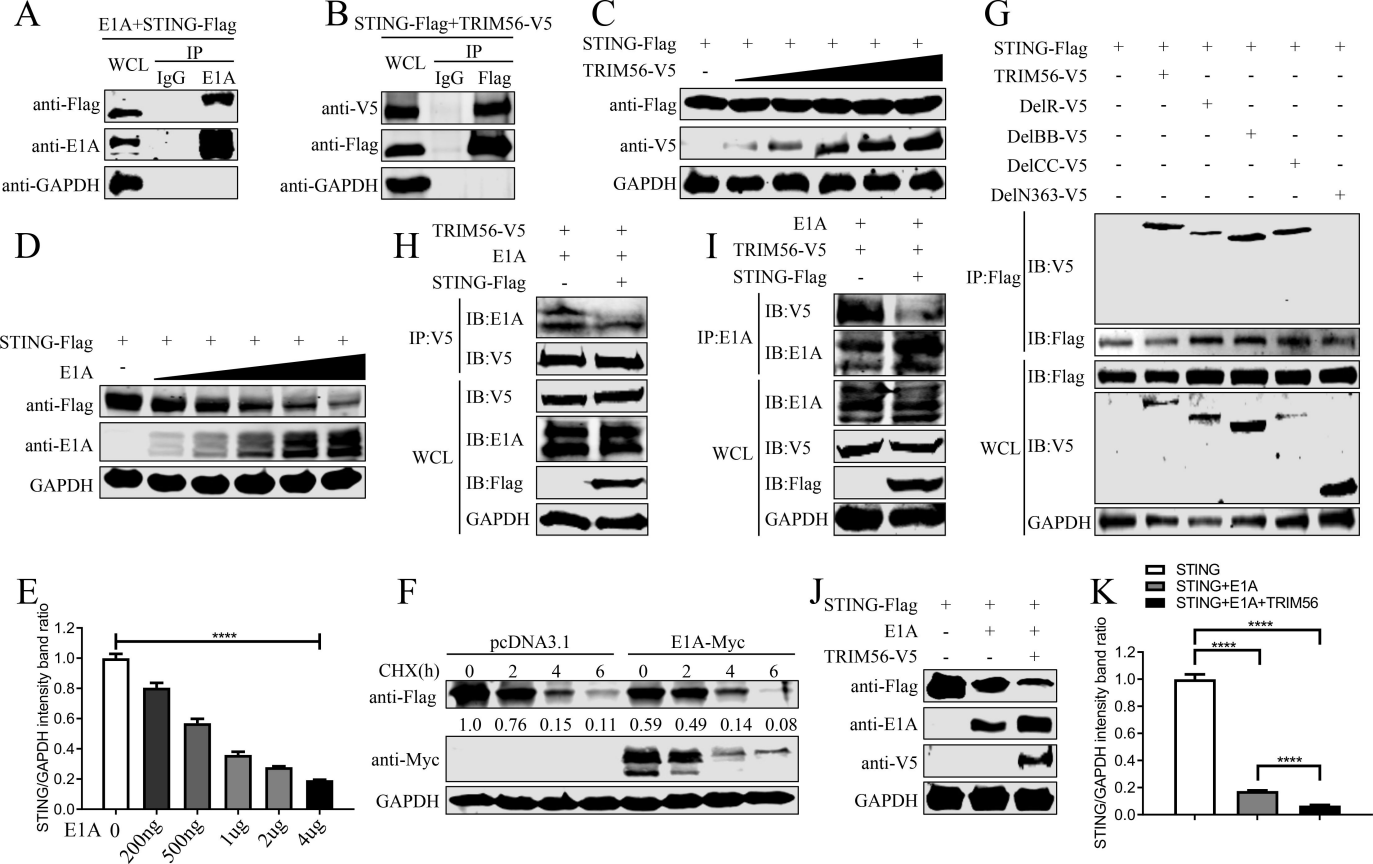
TRIM56 promotes the degradation of STING by E1A. (**A**) Flag-tagged STING and E1A from HAdV-C5 were cotransfected into HEK293T cells for 36 h. Then, the cells were collected, and whole-cell lysates were immunoprecipitated with an anti-E1A antibody or control IgG. Western blotting was performed as indicated. (**B**) V5-tagged TRIM56 was cotransfected with Flag-tagged STING into HEK293T cells for 36 h. Then, the cells were collected, whole-cell lysates were immunoprecipitated with an anti-Flag antibody or control IgG, and western blotting was performed as indicated. (**C**) Gradually increased TRIM56-V5 and Flag-tagged STING were cotransfected into HEK293T cells for 36 h, which were then immunoblotted with anti-Flag and anti-V5 antibodies as indicated. (**D**) Gradually increased E1A and Flag-tagged STING were cotransfected into HEK293T cells for 36 h, after which they were subjected to immunoblotting with anti-Flag and anti-E1A antibodies. (**E**) Quantification of relative STING levels of three independent experiments in (**D**). ****P < 0.0001. (**F**) HEK293T cells were cotransfected with E1A-Myc or pcDNA3.1, together with STING-Flag, respectively. The cells treated with 20 µg/mL cycloheximide for 0, 2, 4, or 6 h and western blot was performed with the indicated antibodies. (**G**) HEK293T cells were transfected with Flag-tagged STING and various V5-tagged TRIM56 mutants for 36 h. The cells were collected, and the whole-cell lysate was immunoprecipitated with an anti-Flag antibody. (**H and I**) HEK293T cells were cotransfected with V5-tagged TRIM56 and E1A, together with or without Flag-tagged STING. The whole-cell lysates were immunoprecipitated with an anti-V5 antibody (left panel, **H**) and an anti-E1A antibody (right panel, **I**), respectively. Western blot analysis was performed with the indicated antibodies. (**J and K**) HEK293T cells were cotransfected with Flag-tagged STING and E1A for 36 h, along with V5-tagged TRIM56. The cells were collected and lysed, and then immunoblotted with the indicated antibodies. Quantification of relative STING levels is shown in (**K**). ****P < 0.0001. At least three independent experiments were conducted.

Given that STING was activated by DNA viruses to induce the production of downstream interferons (24). Previous studies have confirmed that the oncogenic genes E7 and E1A of DNA tumor viruses, human papillomavirus 18 and adenovirus, can inhibit the cGAS-STING pathway (25). Here, we examined the role of STING in the regulation of E1A by TRIM56. Co-IP experiments demonstrated that E1A specifically interacts with STING, and TRIM56 also interacts with STING (Fig. 5A and B).

Considering the relationships among E1A, TRIM56, and STING, we first investigated whether TRIM56 regulates E1A by affecting the protein expression of STING. Dose-dependent experiments revealed that TRIM56 overexpression did not alter STING expression (Fig. 5C), whereas increasing E1A expression resulted in a dose-dependent reduction in STING protein levels (Fig. 5D and E). Cycloheximide chase assays further confirmed that E1A destabilizes STING protein (Fig. 5F). These results together indicate that E1A from HAdV-C5 can significantly reduce the expression of STING, but that TRIM56 does not affect the expression of STING.

Co-IP assays using TRIM56 truncation mutants revealed that STING binds the N-terminal region of TRIM56 (Fig. 5G), similar to E1A. Competitive binding assays showed that STING interferes with TRIM56-E1A interaction (Fig. 5H and I). Western blot analysis suggested that TRIM56 indirectly downregulates STING by stabilizing E1A (Fig. 5J and K). In summary, TRIM56 enhances the expression of E1A from HAdV-C5, which, in turn suppresses STING, antagonizing antiviral immune responses.

### Construction of OAV-TRIM56 recombinant oncolytic adenovirus

**Fig 6:**
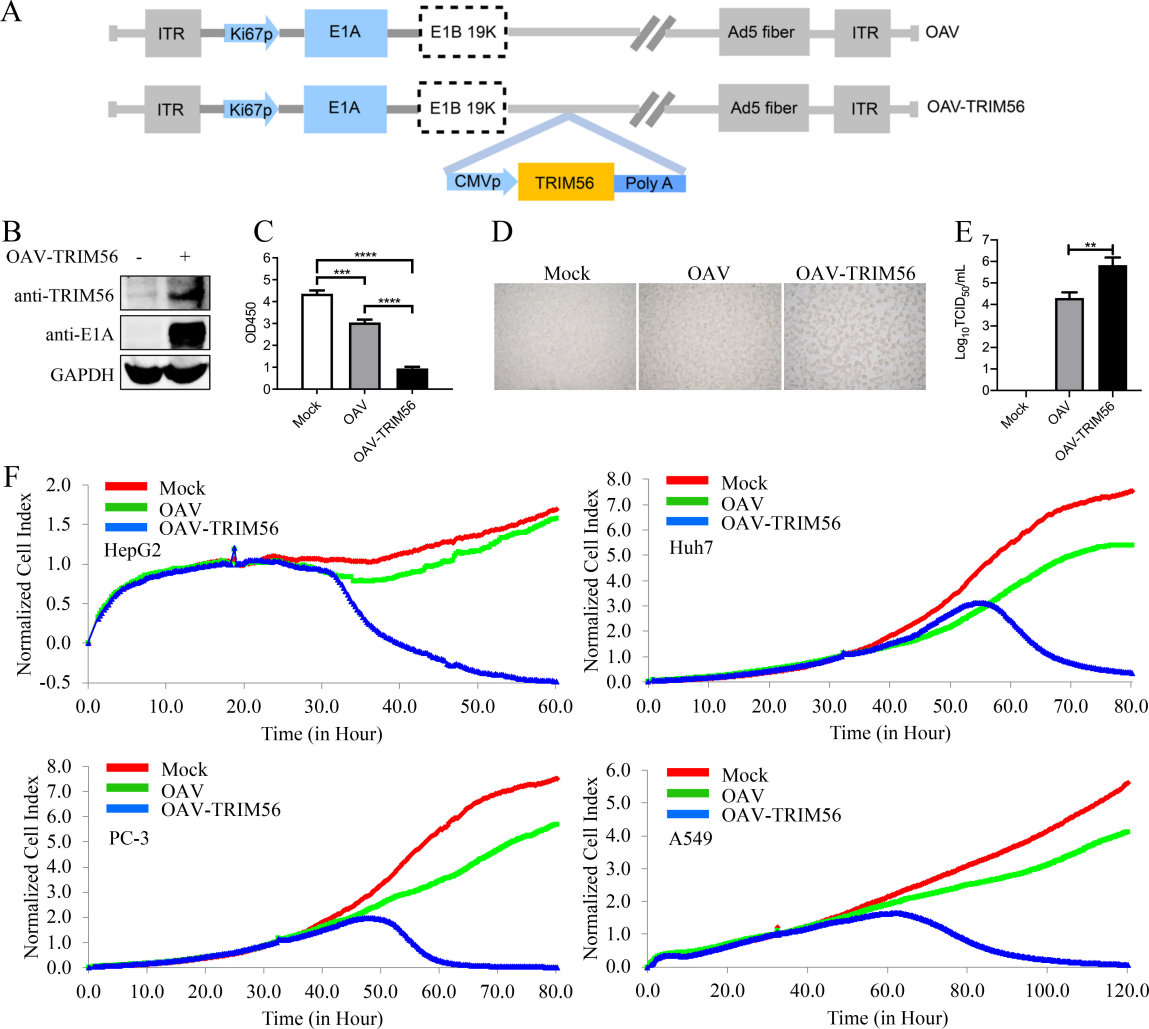
Construction of the OAV-TRIM56 recombinant oncolytic adenovirus. (**A**) Schematic diagrams of recombinant adenovirus shuttle vectors that express TRIM56 with serotype 5 fiber. ITR, inverted terminal repeat; Ki67p, Ki67 promoter; E1A, adenoviral early region 1A; E1B19K, adenoviral early region 1B 19KD; Ad5 fiber, serotype 5 adenovirus (Ad5) fiber. (**B**) The expression of TRIM56 and viral E1A in OAV-TRIM56-infected A549 cells was detected, and noninfected A549 cells were used as controls. At 24 h post-infection, the cells were collected and lysed. The viral E1A protein and TRIM56 were detected with anti-E1A and anti-TRIM56 antibodies, respectively. (**C**) The viability of A549 cells infected with OAV or OAV-TRIM56 was detected via a CCK-8 assay. (**D and E**) HepG2 cells were infected with OAV or OAV-TRIM56 at an MOI of 10. A microscope was used to observe the tumor-killing effect at 36 h post-infection. TCID50 assays were performed to detect the virus titer of the infected cells’ supernatant in (**D**). Scale bar, 200 µm. (**F**) RTCA was performed to analyze the antiproliferative effects of OAV and OAV-TRIM56 on HepG2, A549, Huh7, and PC-3 cells. At least three independent experiments were conducted.

Based on these findings, we constructed an oncolytic adenovirus (OAV-TRIM56) carrying the TRIM56 gene (Fig. 6A). To confirm whether the construction of the OAV-TRIM56 recombinant oncolytic adenovirus was successful, the level of TRIM56 in OAV-TRIM56-infected A549 cells was detected, and noninfected A549 cells were used as controls. The results revealed that OAVs carrying the TRIM56 gene could replicate normally in infected cells and that the expression level of TRIM56 was significantly greater than that in control cells, indicating that the OAV-TRIM56 recombinant oncolytic adenovirus was successfully constructed (Fig. 6B). Similarly, CCK-8 assays revealed that OAV-TRIM56 exhibited stronger cytotoxicity than conventional OAV (Fig. 6C).

Microscopic examination revealed more pronounced cytopathic effects in OAV-TRIM56-infected cells (Fig. 6D). TCID50 assays showed that OAV-TRIM56-infected cells produced more virus particles than OAV-infected cells (Fig. 6E). Real-time cellular analysis (RTCA) experiment further confirmed that OAV-TRIM56 had a significantly stronger oncolytic effect than OAV (Fig. 6F). These results indicate that the OAV-TRIM56 recombinant virus promotes the proliferation of the virus and has a more significant oncolytic effect than the traditional OAV does.

### Effect of TRIM56 on oncolytic activity *in vivo*

**Fig 7:**
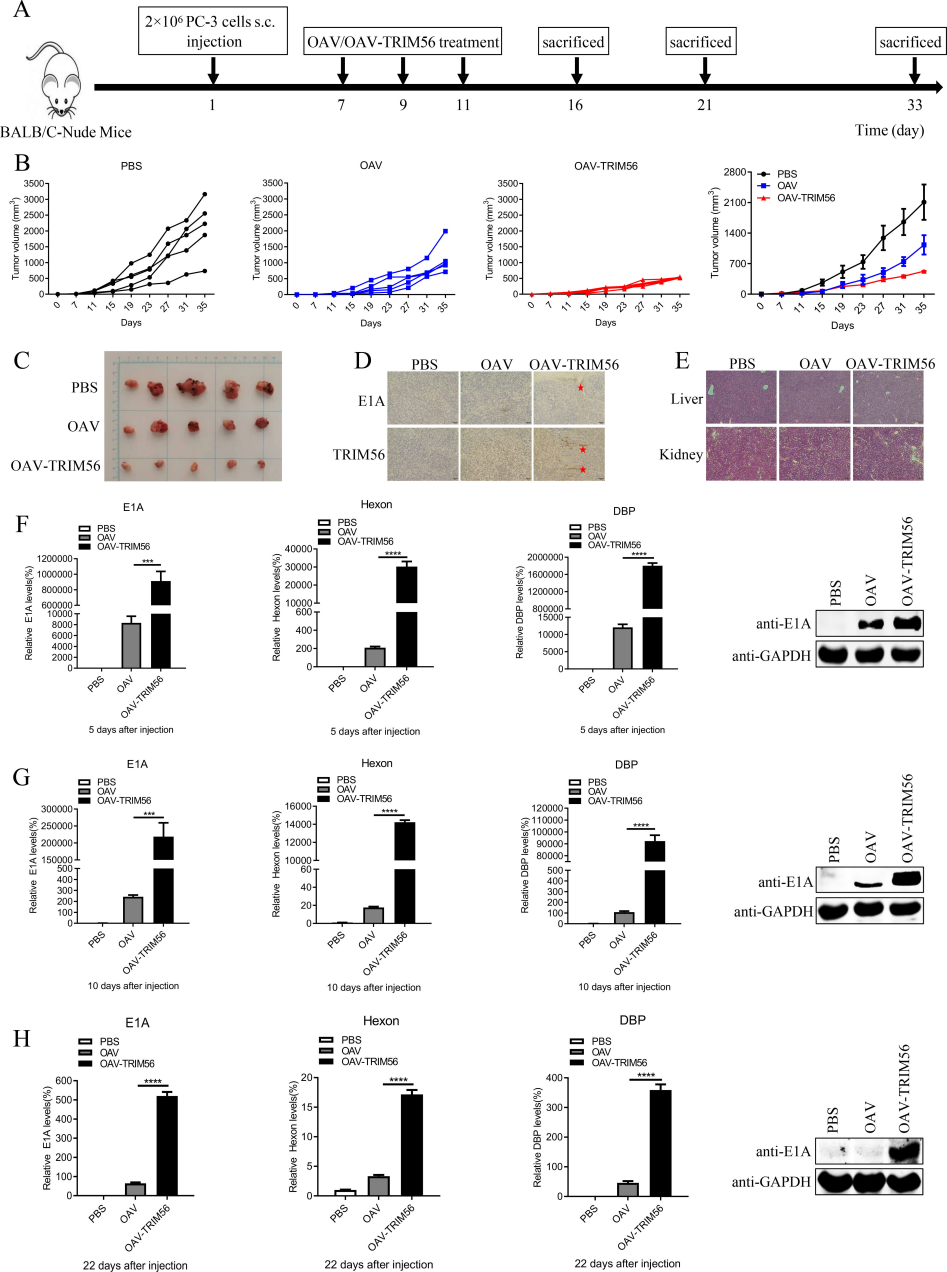
Effect of ectopic TRIM56 expression on oncolytic activity *in vivo*. (**A**) The experimental timeline of the *in vivo* study is shown in the figure. PC-3 cells (2 × 10^6^ cells per mouse; *n = 15* per group) were inoculated into nude mice subcutaneously. The PBS, OAV, or OAV-TRIM56 (1 × 10^9^ PFU) was injected intratumorally once every other day for a total of three times when the tumors reached approximately 50 mm^3^. (**B**) The tumor growth curves of each mouse are displayed. (**C**) The size of the tumor mass in each different group is shown. (**D**) The expression of the E1A viral protein and TRIM56 of OAV/OAV-TRIM56 in the tumor tissues of the mice was detected via immunohistochemistry. Scale bar, 50 µm. (**E**) HE staining was performed to detect the effects of OAV/OAV-TRIM56 on liver and kidney toxicity in mice. Scale bar, 100 µm. (F, G, and H) The expression of the E1A, Hexon, and DBP viral genes in tumor tissues was detected on the 5th, 10th, and 22nd days after three OAV/OAV-TRIM56 injections. The expression of the E1A viral protein in tumor tissues was subjected to immunoblotting with an anti-E1A antibody when the tumor tissues were ground. ***P < 0.001; ****P < 0.0001.

To evaluate the *in vivo* oncolytic activity of OAV-TRIM56, a PC-3 subcutaneous xenograft model was established. Mice were divided into three groups when the subcutaneous tumor diameter was approximately 50 mm^3^. Then, mice were injected intratumorally with PBS, OAV, and OAV-TRIM56 (1 × 10^9^ PFU) every other day for 3 days (Fig. 7A). OAV-TRIM56 treatment resulted in significantly reduced tumor volumes compared to PBS or OAV treatment (Fig. 7B and C).

Immunohistochemistry confirmed higher E1A protein levels in OAV-TRIM56-treated tumors, supporting enhanced viral replication and oncolytic efficacy (Fig. 7D). Moreover, HE staining revealed that the OAV and OAV-TRIM56 did not affect the livers or kidneys of the mice (Fig. 7E). In addition, three mice in each group were sacrificed on the 5th, 10th, and 22nd days after intratumoral injection. Real-time PCR detected the expression of E1A, Hexon, and DBP viral genomes in tumor tissue. The data revealed that increased viral production and E1A viral protein levels were observed in tumors treated with OAV-TRIM56 than OAV-treated tumors (Fig. 7F through H). Collectively, these results indicate that OAV-TRIM56 has a greater antitumor effect on prostate carcinoma *in vivo*.

## DISCUSSION

Many viruses are regulated by host factors during infection, yet the precise mechanisms through which TRIM family proteins influence adenovirus infection remain largely unknown. Our previous studies demonstrated that TRIM35 negatively regulates adenovirus replication, with its knockdown promoting viral replication (26). In this study, we demonstrate that TRIM56 enhances adenovirus infection by directly increasing E1A viral protein expression. To elucidate the mechanism by which TRIM56 regulates adenovirus replication and explore its potential in oncolytic adenovirus (OAV) therapy, we conducted an in-depth investigation. Understanding the fundamental interactions between HAdV and host immunity is crucial for advancing adenovirus-based therapeutic strategies.

Oncolytic viruses (OVs) have gained attention in cancer therapy due to their ability to selectively replicate in and lyse tumor cells while sparing normal tissues. Since 2005, adenoviruses have been the most extensively studied OVs and were among the first used in clinical trials. A key advantage of OAV therapy is its immunogenicity, as OAVs can stimulate antigen-presenting cells (APCs), release tumor-associated antigens (TAAs) and damage-associated molecular patterns (DAMPs), recruit immune cells, induce immunogenic cell death (ICD), and directly lyse tumor cells (7, 10, 27). A variety of antitumor genes, including p53, interferons (IFNs), and granulocyte-macrophage colony-stimulating factors, have been used by various oncolytic viruses to enhance their therapeutic effects and used in basic research and clinical trials (28). Despite their promise, OVs face challenges such as limited transmission in solid tumors, reducing their therapeutic efficacy (29).

To improve OV efficacy, various optimizations have been made, including the incorporation of transgenes such as cytokines, immune checkpoint blockers, T-cell activators, and TAAs (30, 31). The inclusion of PD-1 and PD-L1 blocking antibodies, as well as PD-L1 inhibitory proteins, in oncolytic viruses has led to improved therapeutic outcomes in preclinical mouse models (32, 33). In addition, TAAs and T-cell activators are being developed to improve OV therapy (34). Although these modifications have yielded promising results, they have focused mainly on how to regulate the host response, and little attention has been given to improving OV replication. Efficient viral replication within tumors is a key determinant of successful OAV therapy. For example, the knockout of ICP34.5 in T-VEC improved drug safety while maintaining therapeutic efficacy by reducing neurovirulence (35). We propose that identifying host factors that support OV replication could further enhance their therapeutic potential.

Increasing OV replication results in higher progeny virus release, enhancing intratumoral viral spread and exponentially increasing viral load (12, 36). High E1A expression has been linked to increased adenovirus replication and tumor cell lysis, improving OAV efficacy (37, 38). Here, we successfully engineered a novel oncolytic adenovirus expressing TRIM56 and demonstrated that the OAV-TRIM56 outperforms traditional OAVs in both *in vitro* and *in vivo* models. Experimentally, the locally delivered OAV-TRIM56 exhibited stronger replication and prolonged maintenance of virus titers, effectively preventing tumor progression. Our data support the hypothesis that viral proliferation is increased by effective oncolytic virus particles, which promote the replication of OAVs and ultimately effectively kill tumor cells.

Similarly, previous studies have shown that enhancing the replication of oncolytic HSV-1 significantly improves its antitumor activity (39, 40). For instance, the proteasome inhibitor bortezomib induces the production of HSP90, promotes the nuclear localization of HSV-1 polymerase, and increases viral replication, resulting in a synergistic antitumor effect when oncolytic HSV-1 is combined with bortezomib (41). In addition, inhibition of HDAC6 has been shown to facilitate the transfer of oncolytic HSV-1 to the nucleus, counteracting the antiviral effect of type I interferon and thereby boosting viral replication (37). Our study revealed that TRIM56 promotes HAdV-C5 replication by stabilizing the E1A viral protein and assisting in STING antagonism. The engineered OAV-TRIM56 effectively sustains E1A expression, prolongs OV survival *in vivo*, and demonstrates superior antitumor activity compared to traditional OAVs, validating our strategy of targeting E1A to improve OAV efficacy.

In summary, we identify TRIM56 as a critical host factor that enhances HAdV-C5 replication by stabilizing the E1A viral protein, thereby improving viral replication and transcription. The recombinant oncolytic adenovirus OAV-TRIM56 exhibits enhanced replication and antitumor activity, supporting the concept that targeting host factors to promote OAV replication is a promising strategy for optimizing oncolytic adenovirus therapy in cancer treatment.

## MATERIALS AND METHODS

### Mice

All mice were on a BALB/c-nude background and were maintained under specific-pathogen-free conditions. Male mice, 6 weeks of age, were used for all experiments.

### Cell lines and adenoviruses

The human HEK293, HEK293T, A549, HepG2, Huh7, and PC-3 cell lines were cultured in DMEM supplemented with 10% fetal bovine serum. All the cell lines were routinely tested for mycoplasma contamination, and only mycoplasma-negative cells were used in the study.

All viruses were derived from human adenovirus subtype C serotype 5 (HAdV-C5), including HAdV-C5-GFP (wild-type Ad5 expressing GFP) and OAV (*E1B55K* gene deletion). The HAdV-C5-GFP was constructed by incorporating a GFP expression cassette (CMV promoter +GFP + polyA) into the E1 region of human adenovirus subtype C serotype 5. The viruses proliferated in A549 or HEK293 cells. The tissue culture infectious dose (TCID50) was used to detect viral activity and is expressed as plaque-forming units/mL (PFU/mL) (42).

### Plasmids and antibodies

All plasmids were constructed using cytomegalovirus (CMV) promoter vectors. The full-length E1A and the full-length TRIM56 with a V5 tag were constructed into pcDNA3.1. Various TRIM56 mutant plasmids with V5 tags were generated via PCR mutagenesis. DNA sequencing confirmed that all the plasmids were successfully constructed.

The antibodies used included anti-GAPDH (10494-1-AP; Proteintech), anti-E1A (05-599; Millipore), anti-TRIM56 (25509-1-AP; Proteintech), anti-V5 (V8012, V8137; Sigma Aldrich), anti-Flag (F1804, F7425; Sigma Aldrich), and mouse IgG (A7028; Beyotime). Alexa Fluor 633-conjugated goat anti-rabbit IgG (H + L) and Alexa Fluor 488-conjugated donkey anti-mouse IgG (H + L) were procured from Life Technologies. DyLight 800 goat anti-mouse IgG (H + L) and DyLight 680 goat anti-rabbit IgG (H + L) were obtained from Immunoway.

### Viral replication assay by tissue culture infection dose (TCID50)

A549 cells were infected with HAdV-C5 or HAdV-C5-GFP for 2 hours and harvested at 0, 12, 24, 36, 48, and 60 hours post-infection. Cells and media were frozen at −80°C, subjected to three freeze-thaw cycles, and centrifuged to collect virus supernatants. TCID50 was determined in HEK293 cells. After 7–10 days, the cytopathic effect (CPE) was measured, and the virus titer was calculated (26).

### Recombinant oncolytic adenovirus construction

The OAV used was a recombinant adenovirus with a deletion of the *E1B55K* gene and retention of the *E1B19K* gene, utilizing human adenovirus subtype C serotype 5 as a backbone. In addition, tumor-specific promoter Ki67 was used to drive the adenoviral *E1A*. The OAV-TRIM56 was constructed by inserting the TRIM56 expression cassette into the E1 region of adenovirus. Briefly, the TRIM56 cDNA driven by the CMV promoter was synthesized by Azenta Life Sciences and cloned into the adenoviral shuttle plasmid pZD55 (43), in which the *E1B55K* gene had been deleted. HEK293 cells were cotransfected with pZD55-hTRIM56 (2 µg) and adenoviral cytoskeleton plasmid pBHGE3 (2 µg) (Microbix Biosystems Inc.) via Lipofectamine 2000 (Invitrogen) according to the instructions. HEK293 cells were used to amplify the engineered adenovirus AdV5-ZD55-TRIM56, which is OAV-TRIM56. The virus supernatants were collected when cytopathic effect occurred, and the virus titer was measured via the TCID50 method.

### Construction of A549 cells stably overexpressing TRIM56

Retroviral construct pQCXIN-TRIM56 and the empty vector pQCXIN were transfected into AmphoPack-293 packaging cell line (631505, Clontech) using Lipofectamine 2000. After 48 h of transfection, the virus supernatant of the transfected cells was collected for transduction of A549 cells cultured in six-well plates. Forty-eight hours post-transfection, the transduction was repeated once more. The transduced cells were split and screened in the medium supplemented with 1,000 µg/mL G418. The surviving cells were cloned in a 96-well plate for proliferation, and TRIM56 overexpression was detected by quantitative reverse transcription PCR (RT-qPCR).

### siRNA knockdown

siRNA targeting TRIM56 sense: 5′- CCUUCAAGACCAACUUCUUTT-3′, anti-sense: 3′-AAGAAGUUGGUCUUGAAGGTT-5′; scrambled siRNA sense: 5′-UUCUCCGAACGUGUCACGUTT-3′, anti-sense: 3′-ACGUGACACGUUCGGAGAATT-5′ (Genepharma, Shanghai, China) at a concentration of 20 nM was transfected into A549 cells using the Lipofectamine RNAiMAX transfection reagent (Invitrogen). After 48 hours of transfection, RT-qPCR and western blotting were used to detect the knockdown efficiency.

### Western blotting

Whole-cell extracts were prepared and subjected to reduction electrophoresis on 10% sodium dodecyl sulfate (SDS)-polyacrylamide gels and electrotransfer to polyvinylidene difluoride (PVDF) membranes. The membrane was blocked with 5% skim milk prepared with TBS-T (Tris-buffer containing 0.1% Tween 20) at room temperature for 2 h. After rinsing with PBS, the membrane was incubated with the corresponding primary antibody for 2 h at room temperature and then incubated with an immunofluorescence-labeled secondary antibody at room temperature for 1 h. The results were observed via Odyssey.

### RNA extraction and qRT-PCR

HAdV-C5 was used to infect A549 cells, and the supernatants were collected at different infection time points. Total RNA was extracted via TRIzol reagent. One microgram of total RNA was reverse transcribed via the HiScript II First Strand cDNA Synthesis Kit according to the manufacturer’s instructions. After a 20-fold dilution of the cDNA mixture, qRT-PCR analysis was performed using the SYBR Green qPCR master mix. The expression of GAPDH was used to normalize the relative expression of the target gene. The primers are as follows: *E1A-forward: TTGTCATTATCACCGGAGGAA, E1A-reverse: TCACCCACTGCCCATAATTT; Hexon-forward: GGACGCCTCGGAGTACCTGAG, Hexon-reverse: ACAGTGGGGTTTCTGAACTTGTT; DBP-forward: TAATCAAGCATGGCAAAGGAG, DBP-reverse: AATTTCACTTTCCGCTTCG; Human TRIM56-forward: CCGAGGACATTTTCCTGAAG, Human TRIM56-reverse: AGTTAAGGTCACGCCACCAC*.

### Mass spectrometry

Results of mass spectrometry are shown in Table S1. Sample processing and data analysis of mass spectrometry were performed by Shanghai Applied Protein Technology (Shanghai, China) as described previously (26).

### Mouse experiments

PC-3 cells (2 × 10^6^) were resuspended in cold PBS, and the left abdomen of BALB/c mice was injected with the suspension subcutaneously. The tumor size was measured every other day, and the tumor volume was calculated according to the formula V=(L×W^2^)/2. The widest and smallest diameters of the tumor were represented by L and W, respectively. When the tumor volume reached 50–100 mm^3^ (7 days after implantation), 1 × 10^9^ PFU of OAV/OAV-TRIM56 was injected into the tumor once every other day for a total of three times. The tumor-bearing mice were sacrificed on the specified date after implantation, and the tumors were collected and treated for further analysis.

### Statistical analysis

All the data represent the mean of at least three independent experiments, with standard error of the mean (SEM) indicated by error bars. Two-tailed Student’s *t*-test or analysis of variance was used for statistical analysis, *P < 0.05, **P < 0.01, ***P < 0.005, ****P < 0.0001, N.S., not statistically significant.

## Data Availability

The data supporting the findings of this study are available within the article and its supplemental material.

## SUPPLEMENTAL MATERIAL

Supplemental figures jvi.00041-25-s0001.docx Figures S1 and S2.

Supplemental legend jvi.00041-25-s0002.docx Legend for Table S1.

Table S1 jvi.00041-25-s0003.xlsx Analysis of differential results of E1A proteomics.

ASM does not own the copyrights to Supplemental Material that may be linked to, or accessed through, an article. The authors have granted ASM a non-exclusive, world-wide license to publish the Supplemental Material files. Please contact the corresponding author directly for reuse.
