# Supplementary material for: TRIM56 enhances adenoviral E1A steady state to improve oncolytic adenovirus therapy efficacy
Source: J Virol. 2025 Jun 3;99(7):e00041-25. doi: 10.1128/jvi.00041-25 (PMC12282078; doi:10.1128/jvi.00041-25)
Supplement: Supplemental figures — Figures S1 and S2. [file jvi.00041-25-s0001.docx]

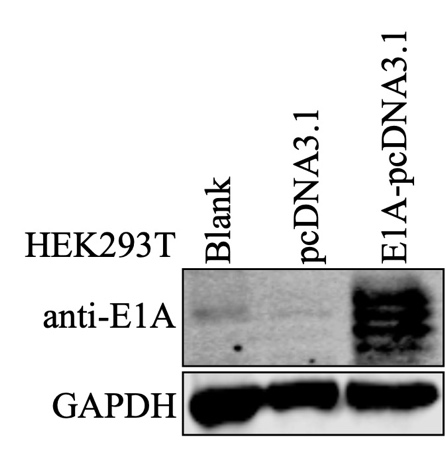


Fig S1 Detection of endogenous E1A protein expression in HEK293T cells. HEK293T were transfected with pcDNA3.1 empty vector and E1A-pcDNA3.1 plasmid (1μg) for 36 h, comparing with HEK293T blank cells, and then western blot detect E1A protein expression.


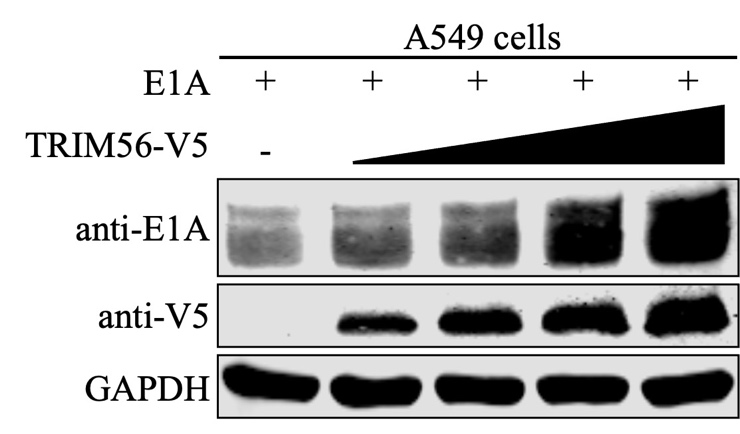


Fig S2 Increased levels of both TRIM56-V5 and E1A were cotransfected into A549 cells for 36 h. Cell lysates were immunoblotted with anti-E1A and anti-V5 antibodies.
