# Supplementary material for: TRIM56 enhances adenoviral E1A steady state to improve oncolytic adenovirus therapy efficacy
Source: J Virol. 2025 Jun 3;99(7):e00041-25. doi: 10.1128/jvi.00041-25 (PMC12282078; doi:10.1128/jvi.00041-25)
Supplement: Supplemental legend — Legend for Table S1. [file jvi.00041-25-s0002.docx]

**Supplemental Table Legend**

The protein information from the mass spectrometry analysis of the E1A overexpression cells and the control cells in Table S1.
